# Nutritional and Behavioral Approaches to Body Composition and Low-Grade Chronic Inflammation Management for Older Adults in the Ordinary and COVID-19 Times

**DOI:** 10.3390/nu12123898

**Published:** 2020-12-20

**Authors:** Jasminka Z. Ilich

**Affiliations:** Institute for Successful Longevity, Florida State University, Tallahassee, FL 32306, USA; jilichernst@fsu.edu

**Keywords:** osteosarcopenic adiposity, low-grade chronic inflammation, COVID-19, precision nutrition, macronutrients, micronutrients, bioactive food components, microbiome, physical activity, chronobiology and circadian misalignment, shift workers

## Abstract

As more insight is gained into personalized health care, the importance of personalized nutritional and behavioral approaches is even more relevant in the COVID-19 era, in addition to the need for further elucidation regarding several diseases/conditions. One of these concerning body composition (in this context; bone, lean and adipose tissue) is osteosarcopenic adiposity (OSA) syndrome. OSA occurs most often with aging, but also in cases of some chronic diseases and is exacerbated with the presence of low-grade chronic inflammation (LGCI). OSA has been associated with poor nutrition, metabolic disorders and diminished functional abilities. This paper addresses various influences on OSA and LGCI, as well as their mutual action on each other, and provides nutritional and behavioral approaches which could be personalized to help with either preventing or managing OSA and LGCI in general, and specifically in the time of the COVID-19 pandemic. Addressed in more detail are nutritional recommendations for and roles of macro- and micronutrients and bioactive food components; the microbiome; and optimal physical activity regimens. Other issues, such as food insecurity and nutritional inadequacy, circadian misalignment and shift workers are addressed as well. Since there is still a lack of longer-term primary studies in COVID-19 patients (either acute or recovered) and interventions for OSA improvement, this discussion is based on the existing knowledge, scientific hypotheses and observations derived from similar conditions or studies just being published at the time of this writing.

## 1. Introduction

The extremely rapid spread of the novel SARS-CoV-2 (COVID-19) virus has created an unchartered, yet urgent, need for investigation into various aspects of its spread, health consequences, management and possible relief. One aspect that needs to be studied includes nutritional, lifestyle and behavioral approaches to improve overall health among the population as a whole, but particularly in those deemed to be at a higher risk of succumbing to the infection, e.g., the elderly, people with obesity, or people with some underlining disease (e.g., cardiovascular, pulmonary). In general, the public has been advised “to eat a healthful diet, exercise, get a lot of sleep and avoid stress” in order to keep healthy. Such general advise comes from the lack of evidence-based research and interventional studies (particularly those based on a long-term follow up) to draw conclusions about COVID-19 infection in relation to other conditions, as well as its prevention or post-disease management. In view of this, medical professionals depend on incidental observations, unforeseen outcomes and detection based on previously established knowledge. They are compelled to give their advice and recommendations based on critical thinking and comparison with other cases of viral infections similar to COVID-19. In this context, it is important to understand that each person might require a different and individualized approach in order to reach his/her optimal health outcomes, but yet some of the generalized knowledge and practice are still important to follow.

As more insight is gained into personalized health care, the importance of personalized nutritional and behavioral approaches is even more relevant in the COVID-19 era in addition to the need for further elucidation regarding several diseases/conditions. One of these concerning body composition (in this context; bone, lean and adipose tissue) is osteosarcopenic adiposity (OSA) syndrome. OSA refers to the simultaneous loss of bone mass and strength (osteopenia/osteoporosis), diminished skeletal muscle mass, strength and functionality (sarcopenia/dynapenia), and gain in adipose tissue (either as overt obesity, or as redistributed fat in abdominal/visceral area, or as infiltrated fat into bone and muscle) [1,2,3,4]. OSA has been studied across the world and associated with poor diet, various metabolic disorders and compromised functionality, including balance, walking speed and handgrip strength, in populations of different backgrounds, sex and age, as recently reviewed in [5,6].

Another critical health aspect is the presence of low-grade chronic inflammation (LGCI) which might run undetected and unresolved for long periods, but which is increasingly recognized as a contributing factor to numerous comorbidities, including OSA [6,7]. In fact, the “cytokine storm” manifested in COVID-19 infection [8] has been suggested to be triggered or at least worsened by the presence of LGCI. In addition to aging, one of the major contributors to LGCI and body composition derangement is the Western-type diet (along with other lifestyle changes) of modern humans. The Western-type diet is highly pro-inflammatory and quite different to the diet our prehistoric ancestors ate when our genome evolved. The most obvious differences are a decrease in energy expenditure and an increase in energy intake via foods low in nutrients but high in energy. [6,7]. To compensate for the decreased intake of nutrient-dense foods, research has expanded on investigating numerous bioactive food components as well as the microbiome in the context of their health benefits and this has been particularly heightened in the COVID-19 pandemic; all addressed below.

Physical activity, or a lack of it, is very much implicated in proper bone and body composition maintenance, prevention of inflammatory processes, and receives as much importance as nutrition in the COVID-19 lockdowns. Further issues intensified by the COVID-19 pandemic include the rise in food insecurity [9] in addition to nutrition inadequacy, along with the increased need for shift and front workers. More people are working long and overnight shifts, leading to wide disturbance in chronobiology. The robust circadian rhythm, set to approximately 24 h intervals, harmonized with sleep/wake patterns, is crucial for the proper functioning and synchronization of all body processes and the regulation of physiological, emotional and behavioral patterns. It is well known that disturbance to the circadian rhythm leads to numerous health problems, including obesity, diabetes, various cancers, and metabolic disturbances [6,10]. Therefore, all these issues are addressed here as well.

The inevitable changes in body composition associated with aging (or other chronic conditions such as diabetes, cancer), osteoporosis, sarcopenia, and obesity, alone or in combination, are implicated in the development of OSA, in addition to LGCI, and are being contemplated as high-risk factors for acquiring or succumbing to the COVID-19 infection. The evidence for cross talk between bone, muscle and adipose tissues and the connection between the immune system and inflammation, the common traits in OSA, have been addressed [1,6,11]. Therefore, OSA syndrome is a good model to examine in the COVID-19 era as it emphasizes the interconnected nature of three body composition components, all modulated by LGCI, stress, nutrition, circadian misalignment, and behavioral, environmental and other lifestyle factors.

Therefore, the overall goal of this comprehensive evaluation of body composition was to address various influences on OSA and LGCI, as well as their mutual action on each other, and present these in the context of typical-normal conditions and also applicable in the COVID-19 pandemic. The following specific issues are discussed: (a) likelihood of OSA metabolic features to influence COVID-19 infection outcomes; (b) nutritional (including bioactive food components and the role of the microbiome) and physical activity recommendations, personalized to help with either preventing or managing OSA and LGCI in general, and specifically in the time of the COVID-19 pandemic; (c) some of the major critical issues during the COVID-19 pandemic contributing to OSA and LGCI, including increased food insecurity and circadian misalignment in shift and front-line workers.

## 2. OSA Metabolic Features May Serve as Predictors for COVID-19 Outcomes

Although OSA could be triggered by various illnesses, the most common cause of OSA is aging, which is also a major contributor to LGCI, as well as numerous other comorbidities, including frailty. In fact, the very biology of aging underlies all subsequent diseases, including a considerably compromised or weakened immune system (immunosenescence) with less responsive T cells, and decreased ability to combat viral infection [6]. Moreover, older individuals, disproportionally affected by both OSA and LGCI, may have inadequate nutritional status, further worsened by possible use of multiple medications. These are suggested as some of the factors why older adults succumb to COVID-19 faster and/or with more severe consequences than younger people [12] and suffer from unproportionally higher death rates, as unequivocally reported so far [13].

Another component of OSA encompasses increased adipose tissue; the obvious phenotype of individuals with overweight and obesity. However, the redistributed fat around visceral organs, as well as infiltrated fat into bone and muscle, is not always observable [1,5]. In this context, it is important to emphasize the adverse influence of visceral/abdominal fat and its secretory molecules in bone and muscle deterioration, as well as in propagation of LGCI. Visceral fat releases multiple inflammatory cytokines, including tumor necrosis factor (TNF)-alpha, interleukins (IL-1 and IL-6), C-reactive protein (CRP), fibrinogens, and lipoprotein-associated phospholipase, all altering inflammatory response, causing not just damage to bone and muscle, but to the whole-body organ systems [14]. These are some of the same inflammatory cytokines creating an immune imbalance in the “cytokine storm” during COVID-19 infection and also one of the reasons why obesity has been emphasized as a potentiating factor in the infection [15], causing significantly worse outcomes, slower recovery (also due to the pressure on diaphragm and heart) and possibly lower response to the future vaccine. Some of these mechanisms are explained in a recent review and meta-analysis [16]. Indeed, based on the recent study in almost 2600 hospitalized patients with COVID-19 infection, those with a BMI >40 kg/m^2^ who were younger than 65 years were more likely to die or be intubated compared even to their older, but not obese, counterparts [17]. Similarly, in a study just published in Circulation, an analysis of the data from the American Heart Association COVID-19 Registry from 88 US hospitals showed that patients with obesity were at higher risk of dying in hospital, more frequently required mechanical ventilation, and were at higher risk for thromboembolism and dialysis, regardless of age, compared to non-obese patients [18].

Infiltration of fat in bone and muscle (another feature of OSA) is impacting their structure and leading to loss of strength and functionality [19]. Additionally, the infiltrated fat leads to heightened LGCI by mechanisms described above and contributes to both bone resorption and muscle breakdown. As reviewed recently, both bone and muscle secretory products influence each other [20]. For example, sclerostin, a marker of bone resorption causes the breakdown of muscle, while myostatin and troponins released during muscle catabolism can damage bones, as well as other organs, contributing to overall health deterioration and frailty. Along this line, it is likely that fat infiltrates the lungs and heart muscles, making them more susceptible to infection and less able to handle viruses such as SARS-CoV-2 [21].

Another feature of OSA, the obvious result of body composition derangement, could be the presence of pre-frailty or frailty. There are several definitions of frailty but, in general, it is characterized by poor health, increased risk of falls, disability, hospitalization and overall rise in morbidity and mortality [22]. As expected, numerous studies examining the outcomes of frail patients affected by COVID-19 have been published and most of them corroborate worse consequences in frail patients. It is worth mentioning a recent multicenter European cohort study that examined COVID-19 infection outcomes in frail patients for whom two different diagnostic criteria for frailty were used. The results showed that regardless of which diagnostic measure was used, these patients had a higher risk for severe COVID-19 infection and more serious consequences. The authors suggest that the new guidance on how to protect individuals with frailty should be established emphasizing that more specific protective and preventive measures are required for them, especially in view of a predicted new COVID-19 outbreak [23]. Indeed, with the possible health consequences associated with OSA and reported so far, including disability, frailty, chronic poor health, increased falls and bone fractures, a lower quality of life, higher nursing home placement and morbidity and premature death [5,6], this syndrome could be the one to assess first in order to detect patients at higher risk.

## 3. Precision Nutrition for OSA and LGCI in Good Times and in the COVID-19 Pandemic

Historically, nutritional recommendations were based on a “one-size-fits-all” principle, with general advice for certain diseases or conditions (e.g., diabetes and cardiovascular disease). Although interest in precision nutrition has been increasing, there are still no large long-term clinical trials showing the efficacy of certain diets/nutrients and/or eating frequencies/times for enhancement of particular health outcomes in an individual or in a small group of people with similar characteristics. In this context, the fundamental role of dietitians-nutritionists would be to recommend a specific diet for a specific individual based on his/her specific condition, built on evidence-based principles. Currently, such precision nutrition data do not exist, except maybe for some cancers. After the recent announcement of the 10-year strategic plan to advance nutrition research, and particularly precision nutrition, issued from National Institutes of Health (NIH)–Nutrition Task Force [24] with earmarked billions of dollars, the situation will likely change.

The strategies to manage or prevent OSA and reduce LGCI will depend on each individual’s age, sex and health status and they may be modified and adapted within precision nutrition/personalized medicine. Currently, some studies are reporting on the nutritional status of older patients affected by COVID-19, as it may serve as a prognostic tool for their recovery. In that line, Racinella et al. examined the nutritional status of over 100 men and women (≥65 years) hospitalized for COVID-19 and reported that their Geriatric Nutrition Risk Index was a significant predictor for survival, even after some confounders were taken into account [25]. Importantly, in one of the earlier studies conducted in Wuhan elderly patients (*n* = 182), 52.7% were diagnosed with malnutrition (via Mini Nutritional Assessment) and also characterized as having diabetes, a smaller calf circumference and lower serum albumin, placing them at disadvantageous outcomes in infection recovery [26]. Indeed, Lidoriki et al. recently presented and justified the hypothesis that both nutritional and functional status in geriatric population may serve as good predictors for COVID-19 outcomes [27].

The nutritional recommendations addressed here are for older adults and denote both those for “normal times” and those for the COVID-19 era, with reference to OSA and LGCI, but not necessarily for patients infected with the disease or those treated in hospital. They are in line with the general guidelines issued for the COVID-19 pandemic by nutrition societies and associations, as well as governments throughout the world, as recently reviewed in [28]. However, they also include and consider specific needs and requirements distinct to OSA and LGCI.

### 3.1. Energy and Macronutrients

Macronutrients (protein, carbohydrates and fat) provide energy to the body; that is, the energy contained within the molecule of the macronutrient is converted into the energy usable by the body with the help of numerous other compounds (enzymes and micronutrients) in well-regulated metabolic processes ([29,30], pp. 63–248).

#### 3.1.1. Energy

Energy intake will depend on the individual’s current weight status and possible need for weight loss or maintenance. It should amount to 25–30 kcal/kg/day, which would be 1750–2100 kcal/day for a 70 kg older individual, where the lower range would enable either weight maintenance or slight weight loss. It is important to keep in mind not just the number of calories, but also the macronutrient distribution [29], as discussed below. In the time of the COVID-19 pandemic and lockdowns, everyday movements and subsequent energy expenditure have been reduced for many, thus energy intake might need to be further restricted to prevent weight gain. Often in such situations, some “comfort foods”—energy high but nutrient low—are consumed, contributing to obesity, weight gain and worsening bone and muscle status. Moreover, aging leads to gradual loss of muscle (2–3% per decade), in a similar fashion to that experienced with bones [31]. Since muscle renders the highest energy expenditure (with the highest basal metabolic rate), even the usual dietary/energy intake might promote weight gain in healthy mid-age to older individuals. Indeed, it is the most common and inevitable reason for weight gain in this population, unless there is a reduction in food and an increase in physical activity [31].

On the contrary, in cases of severe energy restrictions either due to disordered eating (e.g., anorexia nervosa) or some other chronic condition in the elderly, higher than normal bone and muscle loss as well as frailty may occur [6,32]. Moreover, in the time of COVID-19, adequate nutritional status becomes very important in order to balance the immune response to pathogens and other challenges [33], especially when body composition is compromised such as in OSA. In such situations, in order to avoid deficiency of other nutrients (e.g., protein, minerals, and vitamins; see below) possible supplements may be considered. The extreme cases would be patients with high fever and pneumonia (e.g., affected by coronavirus or other viruses). Their basal metabolic rate is heightened and their energy as well as protein needs are elevated. For example, it is well known that fever increases the basal metabolic rate by ~7% for every degree rise in body temperature above 98.6 °F or by ~13% for every degree above 37 °C. These changes should be monitored, possibly even by indirect calorimetry in hospitalized patients, and needs appropriately adjusted. However, such a discussion is beyond the scope of this paper.

#### 3.1.2. Protein

The amount of protein in the diet should amount to approximately 1.0–1.2 g/kg/day or 0.4 g/meal, which is higher than the current recommendations of 0.8 g/kg/day for the general population [34]. The amount is adjusted here to fit the requirements of older people, whose overall food and subsequent energy intake as well as physical activity are typically lower. While higher protein intake is unequivocally recognized as beneficial for muscle (particularly in athletes), the situation is still uncertain for bone and muscle in the elderly. High-protein diets induce excess calcium (and to some extent, magnesium and phosphorus) loss through urine [32], presumably causing higher bone resorption. Another issue of high-protein diets in the elderly has been recently addressed in a study investigating the relationship between high protein intake and circulating advanced glycation end products (AGEs) and their receptors [35]. AGEs form during cooking/frying of food containing sugars, fat and protein, but also endogenously (especially when hyperglycemia is present, such as in pre-diabetic or diabetic patients) [36]. It is well established that AGEs have strong pro-inflammatory and pro-oxidative capabilities, worsening any underlining chronic conditions but particularly contributing to LGCI, which is important to monitor in the COVID-19 era. Brinkley et al. showed that indeed older individuals consuming more protein from both animal and plant sources had higher levels of the carboxymethyl-lysine receptor and the soluble receptor for AGEs, thus questioning the “blanket” recommendations for high protein intake (e.g., >1.3 g/kg) in the elderly [35]. In view of the above, we have also revised our previous recommendations (1.4 g/kg) [4] to the present level of 1.0–1.2 g/kg [5].

Conversely, low-protein diets (even at the level of 0.7 g/kg) interfere with normal bone metabolism, calcium absorption and synthesis of growth factors (e.g., IGF-1), compromising both bone and muscle [32]. The goal should be to achieve ~25% of overall energy intake in a form of good-quality protein, for which meat, poultry, fish, dairy and eggs are the best sources as they have the best bioavailability/digestibility and adequate amounts of all essential amino acids.

It is important to note that the above recommendations could also be achieved with vegetarian or even vegan diets, although requiring some effort because a good combination of complementary protein foods is necessary. Such combinations include pairing of foods such as legumes and grains, or beans and rice (some nations’ staple foods), as well as adding soy proteins and consuming quinoa, hemp, nuts and seeds, which in combination can provide adequate amounts of all essential amino acids, including branched-chain amino acids, important for muscle health. In this context, whey and soy proteins (as supplements) are referred to as being anabolic, easily digestible, and containing adequate amounts of essential amino acids, as well as having immunomodulatory, anti-inflammatory and antiviral properties [37,38]. Moreover, plant protein foods have advantages as they are rich in minerals, vitamins and phytochemicals, they improve gut microflora (see below) and are environmentally sustainable [38].

#### 3.1.3. Carbohydrates

The remaining energy intake should be balanced between carbohydrates and fats in order to include ~40% of energy intake from non-processed carbohydrates, such as whole grains, with very limited simple sugars which should amount to less than 10% of carbohydrate intake. The US National Academies of Sciences, the Institute of Medicine, the Dietary Reference Intakes (DRI) and the World Health Organization (WHO) all recommend higher fiber intake for women and men, with the range of approximately 25–38 g/day [34,39], with WHO recommendations at the lower end. Higher fiber intake has been attributed to having many health benefits in dose response, including supporting weight loss, increasing bowel and digestive health, and lowering risk for cardiovascular disease and diabetes, particularly in older individuals. However, these recommended amounts are harder to achieve in a modern Western diet and particularly in older people with generally lower food, including fresh fruit and vegetable, intake. Surveys throughout the world report a 50% or lower intake of fiber compared to recommendations [39]. Therefore, in addition to increasing plant foods, some pre- or probiotic supplements may be considered as well.

#### 3.1.4. Fat

Dietary fat (triglycerides, cholesterol, saturated, monounsaturated fatty acids and polyunsaturated fatty acids (PUFAs)), should amount to ~35% of energy intake, with <7% saturated fatty acids (found in bacon, lard, butter, fatty red meat and coconut and palm oils) and none or very limited trans fatty acids (found in processed foods within hydrogenated oils, mostly to extend the shelf life). Although dietary fat is usually associated with negative connotations due to its high energy per weight, the focus should be on increasing omega-3 PUFAs (so called “good fats”). Further, “good fat” found in fish and lean meat helps elevate the metabolic rate, obviously crucial in burning fat and fostering lean mass accrual.

Ideally, the diet should include approximately 1 g/day of omega-3 PUFA, the natural metabolic products of the essential, omega-3, alpha linolenic acid [7,40,41]. Alpha linolenic acid metabolizes into the anti-inflammatory eicosapentanoic acid (EPA) and docosahexaenoic acid (DHA). Aside from their strong anti-inflammatory effects, important in the COVID-19 era, many other benefits of omega-3 PUFAs are well established including their role in bone, muscle and adipose tissue health [7,42]. The results of the recent pairwise meta-analysis comparing the effects of EPA and DHA supplementation on proinflammatory markers, including CRP, IL-6, TNF-alpha and adipokines, showed beneficial but not differential effects of any of the omega-3 PUFAs on the markers examined [43]. While it is widely recognized that fatty fish (e.g., salmon, herring, and sardines) are the best sources of omega-3 PUFAs, some plant foods (walnuts, seeds, especially flaxseeds, and chia seeds) also provide good amounts, again reinforcing that people with vegetarian/vegan habits have other options to achieve adequate amounts in their diet. Regarding other aspects of fat intake from plant or animal sources, although some plant foods might be high in saturated fatty acids (e.g., coconut), their intake delivers multiple vitamins, minerals and antioxidants, rendering important health benefits, as well as being environmentally sustainable [38].

### 3.2. Micronutrients

Micronutrients, vitamins and minerals (elements), have been studied for decades and their functions in various systems is well established. However, some micronutrients have received renewed attention because of their roles in immune modulation, their antioxidative properties and beneficial effects observed against viruses, including SARS-CoV-2, in addition to their functions in regulating body composition, as discussed below. In general, the optimal diet should include the balanced consumption of both vitamins and minerals, possibly at or above DRI [34], unless otherwise noted in the case of population excess intake (e.g., sodium and phosphorus). In cases of low intake or overt deficiencies, supplements are advisable.

#### 3.2.1. Vitamins

All vitamins are essential nutrients and humans (as well as animals) depend on food to meet these requirements. Some vitamins can be partially formed endogenously in the body (e.g., vitamins D, K, and B_12_), although not in adequate amounts. They are divided into those that are fat soluble (vitamins A, D, E, and K) and those that are water soluble (the B group of vitamins and vitamin C) ([30], pp. 307–414), [44] but are discussed in alphabetical order below.

- Vitamin A and its active metabolites (retinol/retinal and retinoic acid) are important in the bone remodeling process and both osteoblasts and osteoclasts contain nuclear receptors for retinoic acid. However, it appears that both too high and too low levels of vitamin A could impair bone health in older individuals. High intake and mega-doses of vitamin A were detrimental for bones in postmenopausal women [45], but this was not observed for any of its precursors (e.g., carotenoids, otherwise very strong antioxidants). An earlier study showed the beneficial effect of plasma carotenoids on some functional performance measures (hip flexion, knee extension, and grip strength) in older men and women over a period of six years [46]. In any case, as long as vitamin A is consumed within recommended levels (700 µg/day), it is both safe and beneficial to bone and overall health [32]. Recently, retinoids were shown to reduce the severity of some viral diseases (e.g., influenza and rotavirus) and block the replication of SARS-CoV-2, thus, they are receiving attention and more research is underway [47].
- Vitamins from the B group—B_1_ (thiamine), B_3_ (niacin) and B_6_ (pyridoxine)—are involved in oxidation-reduction reactions as coenzymes for multiple enzymes, as well as in energy and protein metabolism, thus, regulating body composition. Additionally, their deficiency has been connected with impaired neurotransmitter synthesis, although the studies are of a cross-sectional nature. Some studies are just emerging connecting B vitamins in the etiology of sarcopenia [48]. Vitamins B_9_ (folic acid) and B_12_ (cyanocobalamin), both involved in the recycling of homocysteine (toxic to mitochondria and cardiovascular system) to methionine, have been implicated not just in some body composition regulation but also in stress-related mitochondrial damage and impaired neurotransmitter signaling [6]. However, their action on bones [49] and muscle is inconclusive or non-existent.
- Vitamin C, best known for its antioxidative properties, has also been investigated in diseases such as cancer, cardiovascular disease, and cataracts, as well as bone and muscle health. Vitamin C is required for collagen crosslinking, and its deficiency leads to the weakening of the collagenous structure in bone. Its deficiency also increased relative risk of hip fracture, possibly due to increased free radical generation and bone resorption, as reviewed earlier [32]. In a recent study, higher dietary and circulating levels were correlated with higher muscle mass in older men and women [50]. In short, vitamin C, along with other antioxidant vitamins, protects skeleton and muscle from oxidative stress. Vitamin C also regulates the immune system by increasing the production of interferons, potent viral inhibitors [51], and thereby receives much attention as a potential protector, not just from common cold and flu but potentially from COVID-19 infection as well.
- Vitamin D has been studied extensively throughout the years. In addition to its undisputed involvement in bone metabolism/calcium absorption, higher serum levels of 25-hydroxyvitamin D (25OHD—the circulating metabolite) have been associated with improved balance, lower fall risk, and increased muscle performance, while its deficiency is associated with sarcopenia, as reviewed earlier [52]. Recently, vitamin D has received special attention due to its possible beneficial effects in COVID-19 infection. The intake of approximately 1000 IU/day of cholecalciferol is recommended to keep the serum levels of 25OHD at ≥30 ng/mL (considered adequate) in older individuals. In the time of COVID-19, some researchers recommend a brief intake of 10,000 IU/day for a quick rise in serum 25OHD, followed by 5000 IU/day for maintenance to be taken by high-risk individuals [53]. These recommendations are much higher than the current DRI of 800 IU for individuals over 70 years [34] but could be justified. A recent study reported a significantly higher risk for being infected with COVID-19 in patients with inadequate serum 25OHD compared to those with adequate status [54]. These beneficial effects could probably be attributed to vitamin D’s role in enhancing both innate and adaptive immunity and lowering inflammation (and probably a “cytokine storm”) as it stimulates the production of anti-inflammatory cytokines (e.g., IL-3, IL-4, and IL-10) but inhibits the production of proinflammatory cytokines (e.g., TNF-α, IL-2, and IL-9) [55]. Additionally, evidence shows that vitamin D could reduce respiratory infections (including influenza) overall, as it induces cathelicidins and defensins, which lower viral replication and improve the lining of lung tissue [53]. However, randomized controlled trials are still needed to confirm such effects.
- Vitamin E is a potent peroxyl radical scavenger, particularly protecting PUFAs within phospholipids of cell membranes and plasma lipoproteins, acting as a lipid oxidation chain-breaking antioxidant and arresting further lipid auto-oxidation and subsequent formation of free radicals in the body. Vitamin E also shows immunomodulatory effects [56]. Together with selenium, it is crucial in glutathione antioxidative recycling [32]. However, as with beta carotene supplementations in earlier epidemiological studies investigating cancer risk prevention, the results were contradictory [57]. There is not much evidence of vitamin E involvement in bone. Preclinical studies show benefits on myoblast proliferation, differentiation, survival, membrane repair, mitochondrial efficiency, muscle mass, muscle contractile properties, and exercise capacity [58]. In a recent study, higher intake has been associated with better lean tissue indices, in addition to vitamin C and carotenoids [59].
- Vitamin K is best known for its role in regulating the vitamin K-dependent clotting factors involved in blood coagulation, including prothrombin and other proteins containing gamma-carboxyglutamic acid (Gla) residues. Its role in bone metabolism involves carboxylation of osteocalcin (bone turnover marker), which is a protein containing three Gla residues and necessary for bone mineralization in this carboxylated form in order to bind calcium ions within the hydroxyapatite crystals [32]. High plasma levels of vitamin K were associated with muscle strength, larger muscle mass, and higher physical performance in some observational studies, although the mechanism is not known [60]. However, in the COVID-19 era, another protein, known as matrix Gla protein (MGP), containing five Gla residues and dependent on vitamin K to be carboxylated, has received attention. MGP is present in vascular tissue and, through its ability to bind calcium ions, prevents vascular calcification and elastic fiber degradation, both detrimental in COVID-19 infection [61].

#### 3.2.2. Minerals

Minerals are inorganic compounds and are present in the body in small, sometimes trace, amounts (thus, some are referred to as trace minerals/elements), but they are essential for numerous and varied functions—as structural components (e.g., bones, teeth), as electrolytes, and as part of normal cellular activities and metabolic pathways, being the obligatory cofactors for many enzymes ([30], pp. 425–542). Among minerals, calcium and magnesium have been investigated the most in relation to bone, muscle and adipose tissue involvement [32], but no less important is adequate intake of trace minerals, copper, selenium and zinc. In addition to their multiple roles in the metabolic processes of bone, muscle and adipose tissue, they improve the antioxidative capacity of mitochondria in repairing DNA damage and reducing inflammation and stress [6]. However, their antioxidant capacity is not direct as they act through the endogenous antioxidant system and serve as cofactors for several antioxidant enzymes, including superoxide dismutase, glutathione peroxidase and catalase.

- Calcium and magnesium are best known for their roles in bone metabolism and muscle health and the former, along with dairy products, is also implicated in weight loss/maintenance by attenuating adiposity [62]. Based on national surveys, calcium and magnesium are still at a lower or marginal intake in the US population, even when supplements are taken into consideration [44]. An abundance of literature is available for the beneficial effect of calcium on bone and weight [32,62] and the role of magnesium in muscle [63] and adipose tissue through its regulation of insulin sensitivity and serum glucose [64,65]. Therefore, these functions will not be elaborated on here. However, magnesium’s roles in regulating inflammatory response, in curbing pro-inflammatory cytokines and diminishing systemic inflammation [66] are receiving much attention in the COVID-19 pandemic, particularly in individuals with obesity and diabetes, as both diseases are associated with magnesium deficiency [65].
- Copper is mostly accumulated during growth and deficiency is rare as it is present in nearly all foods. Because copper influences collagen maturation, it could affect bone composition and structure. The enzyme lysyl oxidase is a copper-containing enzyme that catalyzes crosslinking of lysine and hydroxyproline in collagen, contributing to the mechanical strength of collagen fibrils and its deficiency has resulted in decreased bone strength in animal models [32]. Copper also has a prominent role in immunoregulation and neutralization of reactive oxygen species, but any possible copper supplementation has to be carefully administered due to its toxicity and pro-oxidative properties when in excess [67].
- Selenium and its importance in human health has been studied since the 1970s and as it is abundantly present in soil around the globe, except in some isolated areas (China and New Zealand), there was not much concern about its deficiency until greater interest in its antioxidative property emerged in the COVID-19 pandemic. Glutathione peroxidases (GPs) are antioxidative enzymes which catabolize hydroperoxides and other oxidative species and they all use selenium as a cofactor [32]. In selenium deficiency, the action of GPs was greatly diminished and was associated with increased cardiovascular (CVD) incidence, some cancers and overall mortality. Additionally, selenium levels are usually reduced in obesity, with the subsequent reduction in expression of selonoprotein genes, further diminishing antioxidative properties and the immune response [68]. A new meta-analysis examined the role of selenium with and without antioxidant mixtures for the risk of CVD, cancers and mortality. The authors included studies which used a combination of antioxidant mixtures (e.g., two or more of the following: vitamin A, retinol, beta-carotene, vitamin C, vitamin E, zinc, and copper) as composite entities and compared them with those where selenium was added. The results revealed that the inclusion of selenium in antioxidant mixtures showed better protection and risk reduction than just antioxidant mixtures without selenium [69], relevant in the COVID-19 era when antioxidants are in focus to reduce inflammation.
- Zinc has received special attention in the COVID-19 era for its anti-inflammatory and antiviral roles [70]. Indeed, a recent study showed that the antiviral agent chloroquine (used in COVID-19 treatment, although disputably) increases zinc transfer into the cells [70]. Among numerous catalytic, regulatory and structural functions of zinc, its role in highly proliferating human cells, such as those of the immune system and intestinal tract, is critical. An earlier review elaborated on the mechanisms of zinc’s beneficial effects in chronic intestinal inflammatory disorders, such as celiac disease, Crohn’s disease and inflammatory bowel syndrome [71]. A recent preliminary study from Spain showed that among ~250 older patients admitted to the hospital with COVID-19 diagnosis, those who had lower serum zinc levels upon admission in hospital had a significantly higher mortality rate and worse outcome compared to those with normal zinc serum levels. Even after adjusting for confounders such as age, sex, illness severity and treatments received, every unit increase of serum zinc lowered the odds of dying by 7% [72]. Regarding body composition, since ~90% of zinc in the human body is located in muscle, bone, skin and hair, it was no surprise that zinc deficiency and/or excessive loss in urine resulted in impaired bone formation, as well as in muscle and connective tissue metabolism, as reviewed earlier [32].

Among other minerals, iron along with phosphorus and sodium should be reduced (except in severely sick patients, for the latter two) and kept in check [6]. All national surveys show that the US population consumes excess amounts of both phosphorus and sodium because they are abundant in all foods and added to soft drinks and processed foods, respectively.

- Iron is a mineral that plays an important role in bone formation, mostly acting as a cofactor for enzymes involved in collagen synthesis. Some earlier studies showed reduced bone breaking strength in iron-deficient rats, suggesting that iron deficiency may play a role in bone fragility. Similarly, as a component of hemoglobin, it is crucial for proper muscle functioning and oxygen distribution throughout the body [32]. Lactoferrin, the iron-binding glycoprotein, is the iron transporter (thus belonging to the transferrin family) and has antimicrobial (particularly in infants as it is in milk) and both anti-inflammatory and immunomodulatory activities [73]. However, iron should be restricted in the diets of elderly individuals. While some children, pregnant and child-bearing-age women might suffer from iron deficiency or even anemia, and young men have high requirements, that is not the case with older women and men. Since the requirements are lower, the dietary recommendations for older women are reduced to just 8 mg/day, compared to 18 mg/day for younger women [34]. A recent study revealed a strong inverse causal connection between iron in blood (dependent on iron intake) and aging processes, by analyzing genetic data in over one million people [74], explaining earlier findings that high blood iron increases the risk for cardiovascular and some other diseases. Additionally, it was established that iron has pro-oxidative properties and higher blood levels promote oxidative stress and mitochondrial damage, as well as sustain pathogens leading to various infections [74], which is particularly important to monitor in the COVID-19 era.
- Phosphorus is an essential mineral and a major component of bone crystal, hydroxyapatite, as well as crucial for muscle work as part of creatine. However, there is a concern that excessive amounts consumed by modern humans may be detrimental to bone and overall health. For example, a rise in dietary phosphorus increases its serum concentration, producing a transient fall in serum ionized calcium, resulting in elevated parathyroid hormone secretion and potentially bone resorption. A frequently raised issue is the potential adverse effect of the consumption of carbonated beverages affecting bone and muscle health either due to phosphoric acid (added as acidulant and the resulting acid load) or replacement of milk and other more nutritious beverages [32].
- Sodium is known to increase urinary calcium excretion. A well-established positive relationship between urinary sodium (reflecting sodium intake) and urinary calcium excretion was shown in both animals and humans. As a determinant of obligatory calcium loss in urine, sodium has also been shown to cause bone loss in animals (especially at low Ca intakes). However, there are only a few studies examining its effect on bone mass in humans and the findings are inconclusive [75]. The interaction between calcium and sodium is even more important when considering the trends in intakes of each: intake of calcium is typically lower than recommendations, while intake of sodium remains consistently high. However, whether habitual salt excess decreases bone mass and increases the risk for fracture is still not established [75].

### 3.3. Bioactive Food Components

Bioactive food components, also called various other names (nutraceuticals, functional foods, flavonoids), and their actions have been intensely investigated based on numerous purported benefits on health, attributed mostly to their antioxidative capacity. However, these compounds are still under a lot of scrutiny with regard to their definition, different names, precise actions and benefits [76]. For example, the names nutraceuticals and functional foods are in some instances used interchangeably and in others a clear distinction between them is stated. Different countries also include different components under the bioactive term, but the highest disagreement arises from the inability to clearly show their health benefits in some instances and justify different health claims [76].

Regarding bone, the most promising compounds have been those with mildly estrogenic properties such as isoflavones (genistein and daidzein), originating mostly from soybeans and some other legumes. They showed some mild benefits in reducing bone resorption and no side effects with moderate consumption [77]. Among other bioactive components affecting bone, interventions with dried plums have resulted in some promising effects on bone both in ovariectomized rodent models and postmenopausal women [78]. Dried plums are rich in fiber, polyphenols such as chlorogenic acids, as well as some minerals and vitamins that are known to benefit bone; therefore, the benefits of dried plums are probably the result of the synergistic effects of many constituents. Some experimental evidence investigating the effects of various polyphenols (flavones, isoflavones, and anthocyanins) in muscle, has shown limited beneficial effects in reducing free radicals and improving mitochondrial oxidative capacity [79].

Recently, the mechanism of some of dietary phytochemicals (e.g., flavonoids, organosulfur compounds, phenolic acids, and oligosaccharides) previously shown to have osteoprotective and/or gut-beneficial functions were evaluated [80]. The researchers reported that some of these phytochemicals influence T cell differentiation and activation both within gut and bone, suggesting their mediation of bone metabolism via the gut–bone axis. The authors suggest that the effects of those phytochemicals in the intestinal tract may have prebiotic effects on immunomodulating probiotics [80] which could be another connecting factor between the immune system and bone health. (See more on pre- and probiotics in the Microbiome section.) The implications of long-chain polyunsaturated fatty acids (e.g., conjugated linoleic acid, alpha-linolenic acid, and omega-3 fatty acids) as functional foods have also been studied, showing various benefits on bone, muscle and fat tissues [40,41,81]. However, as far as lean and adipose tissue, most investigations have been performed using supplements for muscle enhancement and/or weight loss and they will not be discussed here.

Within the studies investigating the role of bioactive components in inflammation, polyphenols (isoflavones and anthocyanins), as well as quercetin, catechin and resveratrol showed the strongest benefits. During some earlier viral outbreaks (e.g., severe acute respiratory syndrome (SARS) and MERS), it was shown that some bioactive food components (primarily from plant foods) could prevent viral replication as well as over-reaction of the immune response which may lead to a “cytokine storm”. The mechanism is attributed to the ability of these compounds to block cell receptors for angiotensin-converting enzyme 2 (ACE-2) and/or dipeptydilpeptidase 4 (DPP-4) which coronaviruses use for entry into the cell [82]. Among some of the most studied of such compounds are curcumin (found in turmeric), quercetin (found in onion), and sulforaphane (found in broccoli). They have all shown promising results in curbing the actions of various viruses, including influenza, SARS, and ZIKA and future results from studies on SARS-CoV2 are expected to give further insight into those mechanisms.

It is important to note that in addition to some of the proven or claimed benefits of bioactive food components in various cases and situations, their uncontrolled consumption is not advisable as it might lead to serious side effects with other food constituents or interactions with medications, triggering unexpected pharmacological effects, including adverse drug events [83], or there might be no effect at all (in which case it would be a waste of money), although such studies are less frequently published. Therefore, a balanced diet rich in antioxidant foods, such as fruits, vegetables, legumes, whole-grain cereals, nuts and seeds could be the best approach to obtain all essential minerals and vitamins, as well as other phytochemicals and bioactive food components to maintain body composition, reduce inflammation and cope in the uncertain times of the COVID-19 pandemic. One specific example could be to follow Mediterranean dietary patterns (high amounts of monounsaturated fatty acids, omega-3 PUFAs, fish, vegetables, fruits and nuts; moderate amounts of dairy foods, meats and wine; and limited amounts of saturated fatty acids), which was shown to benefit both bones and muscle [84], modulate body fat and reduce inflammation, particularly in older individuals. Along these lines, the U.S. News and World Report ranked the Mediterranean diet as the best overall diet for 2020 [85].

## 4. Microbiome

As we know so far, SARS-CoV-2 is an enveloped RNA coronavirus that attaches to the ACE-2 receptors primarily seen in the lungs, although also present in other tissues, including the enterocytes of the digestive tract and thus may disrupt normal intestinal flora, leading to different gastrointestinal symptoms and treatment challenges. Evidence has also shown the presence of SARS-CoV-2 RNA remnants in feces, indicating its presence in the gut [86]. Moreover, a pilot study in COVID-19 patients of various degrees of severity showed that all of them had significant alterations in the fecal microbiome compared to healthy controls [87]. Their fecal shedding was characterized by a higher presence of opportunistic pathogens, which persisted even after the virus was cleared. Another cross-sectional study compared fecal microbiota of patients with COVID-19 with those with influenza A(H1N1) and healthy controls. The fecal microbiome of COVID-19 patients had the highest number of pathogens and the lowest number of beneficial bacteria and was different to both influenza patients and controls [88]. Based on the biomarkers identified in each cohort, the authors proposed possible identification tools for distinguishing patients from healthy controls.

Many nutrients and food components as well as environmental factors affect intestinal flora and the microbiome and subsequent health outcomes, especially in the elderly. It was postulated that changes in the microbiome occurring in older individuals when they transfer to long-term facilities and are exposed to different food and environment (often less optimal) might worsen their body composition, increase LGCI and quickly result in OSA [89]. Although addressing the microbiome and all its health outcomes is beyond the scope of this paper, some important new findings providing more insight into the complex food–gut–body composition axis relating to COVID-19 are worth mentioning.

It was shown that the amino acid, glutamine, could influence the microbiome by increasing its resistance to stress and improving overall enterocyte health, particularly in critically ill patients [90]. Glutamine was also found to be beneficial in obese and overweight individuals by improving the diversity of their gut microbiota [90], in skeletal muscle (together with arginine) for reducing inflammation and in bone homeostasis for regulating mesenchymal stem cell (MSC) osteoblastogenesis [91]. A new review addressed the possible mechanism of the gut–muscle axis in sarcopenia (based on animal and human studies), inferring that certain gut microbes produce compounds that regulate muscle metabolism in either a positive or a negative way [92]. In addition to dietary intake, the authors reviewed various other factors contributing to mutual influences on the gut–muscle axis, including inflammation, metabolic resistance, mitochondrial dysfunction and modulation of gene expression. In view of the possible therapeutic options, nutritional (e.g., high-protein diets, supplements with various nutrients, active compounds and probiotics), fecal microbiota transplantation, as well as exercise were explored, and reported different levels of success [92].

Regarding probiotic and/or symbiotic (the latter is the addition of prebiotics to probiotics) supplementation and treatment, no primary research is available for COVID-19 or other coronaviruses-affected patients with mild to moderate symptoms [93]. A recent review addressed the role of probiotics as an adjuvant therapy in COVID-19 infection and other viral and inflammatory diseases [94]. Although the role of probiotics in the immune system is not fully understood, it is postulated that they have the ability to regulate and stimulate the immune system, attenuating immune dysfunction in both chronic and acute inflammatory diseases. Ingestion of high levels of probiotics was shown to reduce some and promote the release of other cytokines, as well as interferon-gamma, all possibly due to stimulation of intraepithelial lymphocytes [94]. Some evidence exists for the positive outcomes of symbiotic treatments in other critically ill patients on mechanical ventilators and with severe pulmonary diseases. There are also some clinical trials in COVID-19 patients currently underway which might soon provide more insight into the issue [93]. The lack of evidence is of no surprise not just because of the novelty of some coronaviruses but also due to the complex actions of both pro- and prebiotics regarding their viability, specificity (strain/substrate), dose, and duration in the context of providing possible beneficial immunologic, metabolic and protective effects [94].

## 5. Precision Exercise and Physical Activity for OSA in Good Times and in the COVID-19 Pandemic

Regarding exercise programs, activities and movements should be developed based on the individual’s preferences and abilities [4,5,95]. While following the official exercise regimens (e.g., for weight loss, muscle strength, balance improvement, or overall well-being) could serve as a general guideline, the regimen depends on each individual’s condition, abilities and state of mind.

Since OSA is a complex condition involving bones, muscle and adipose tissue, we recommend a comprehensive exercise program, as addressed earlier [4]. Emphasis is on the improvement of balance and a reduction in falls, as well as LGCI. The program could include all or some of the following options: aerobic, resistance, endurance, weight bearing, strength, flexibility, and balance in the duration and frequency found most achievable. Although the general official recommendations are 60 min of some kind of structured physical activity 5 days/week to realize the best benefits, performing any kind of exercise and in a duration to fit individual’s abilities and preferences could be beneficial to bone, muscle, body weight and body fat, and proinflammatory cytokines, as well as result in improvements in balance, flexibility, cognition, and reduction in falls. Consistency and regularity are especially important in the COVID-19 era when many gyms are closed, and structured/guided exercise are limited. In this situation, walking/jogging or some other self-guided activities, either outside or inside, could be beneficial and enjoyable. An earlier study in older women showed that even habitual physical activities, such as gardening, stair climbing, and household chores were associated with lower weight and better bone health outcomes [96].

Additionally, exercise such as yoga, Pilates, and tai chi have shown similar benefits but perhaps to a slighter degree, when considering body composition outcomes [95]. These types of activities are safe and popular as stress and anxiety reduction strategies, as shown recently for yoga [97]. Similarly, a recent meta-analysis found an over 30% reduction in falls and fall risk in older individuals who were engaged in dance-based mind–motor activities [98]. Such activities (e.g., ballroom and folk dancing), incorporate upright movements, instructions, choreography, inner rhythm and breathing and social interactions. In the time of COVID-19, such activities can be easily found on YouTube or Zoom and since they are enjoyable, adherence is higher. More studies examining these exercise forms will provide evidence regarding their benefits for the prevention and treatment of OSA, and a reduction in LGCI and stressful states of mind.

## 6. Critical Issues to Consider in the COVID-19 Pandemic

### 6.1. Food and Nutrition in the US Food System Contributing to Increased COVID-19 Risk

There are two main issues regarding food and nutrition within the United States Food System which are highly exacerbated in the COVID-19 pandemic. One is food insecurity, referring to the limited or uncertain access to any food due to low income and other socioeconomic circumstances and another is nutrition inadequacy, referring to limited or inadequate nutrients, either by choice, by lack of knowledge or by lack of availability of healthy foods. Both conditions worsen overall health and may contribute to OSA and LGCI. Most importantly, both conditions go together with obesity and they overlap when the availability of healthy foods is in question, whether healthy foods are not consumed because of choice or unawareness or unavailability. Based on the most recent report [99] analyzing data from the National Health and Nutrition Examination Survey (NHANES), food insecurity doubled from 1999 to 2016 in all racial/ethnic groups and both sexes, but was more dominant in individuals who also had generalized and/or abdominal obesity. According to this and other reported studies, it is predicted that food insecurity during this disruption in the economy and health care will increase further and contribute to increased morbidity and mortality in the general population long after the COVID-19 pandemic [9]. This is also true for nutrition inadequacy, which is accompanied with an increased risk for numerous comorbidities, obesity being the most common, but also for osteoporosis and sarcopenia, as well as for heightened inflammatory processes. Obviously, more attention should be given to monitoring and developing adequate policies and programs to diminish both food insecurity and nutrition inadequacy in order to curb not just OSA but many other comorbidities as well and prevent their convergence with COVID-19 infection.

### 6.2. Circadian (Chrono) Rhythm and Chrononutrition/Physical Performance

It is beyond the scope of this paper to discuss chronobiology and all the negative consequences of circadian misalignment. However, in brief, on orientation and tentative day/night time events, the following are some of the main physiological and metabolic/hormonal changes during a normal diurnal period (roughly expressed in military time): (a) between ~21:00 and 22:00 h, melatonin secretion rises (night period) and intestinal peristalsis ceases; (b) at ~2:00 h, the deepest sleep ensues; (c) between ~7:00 and 10:00 h, melatonin secretion ceases (day time), intestinal peristalsis resumes and the greatest alertness follows; (d) at ~15:00 h, the lowest alertness occurs; (e) between ~18:00 and 19:00 h, the highest blood pressure and body temperature takes place; (f) at ~21:00 h, the cycle repeats [10]. Based on this conventional (normal) circadian rhythm, all other body processes are regulated, including digestion, absorption/metabolism of nutrients and outcomes of physical activity; in the disrupted chrono rhythm, physiological, metabolic and emotional consequences are numerous.

#### Shift Workers and Front-Line Respondents

It has been known and widely accepted among dietitians and nutrition professionals that having a regular meal schedule, eating small portions, chewing slowly, avoiding distractions while eating and having a mindful approach to food are the best practices for behavioral modification to achieving long-term weight maintenance [100]. Such behaviors are often not possible for shift, essential and front-line workers whose numbers, as well as the need for them, are at the steep upsurge in the COVID-19 pandemic. Other than trying to establish regularity in everyday activities when possible or optimize the work schedules of shift workers depending on their chronotypes (morning/evening), there are some general nutritional and behavioral practices that might attenuate adverse consequences of circadian misalignment, as recently reviewed in [101,102]. In this context, intermittent fasting (IF) and time-restricted eating (TRE) are receiving more attention, not just from the perspective of weight loss, but also as ways to achieve some other metabolic benefits, especially important in shift and front-line workers in whom adjusting the timing, frequency, and kind of food consumption is critical. For example, TRF may prevent excessive body weight gain, glucose intolerance, hepatic steatosis, and LGCI otherwise induced by high-fat diets [103]. In addition, TRF may protect against the high-fat-diet-induced dulling of clock genes and subsequently prevent several metabolic dysfunctions by salvaging gene expression of rhythmic peripheral clock [103].

It is important for shift workers to differentiate between their rest (sleeping) phase and active (wake) phase, although those might not align with the actual day/night times. It was shown in mice that muscle mass decreases with feeding in the rest phase due to inactivation of IGF-1 signaling [104]. Therefore, TRF during the active phase could be effective for the maintenance of several biological functions, while eating during the rest phase might diminish muscle mass and other body functions.

Briefly, within chrononutrition, the most studied aspects regarding the timing of a meal consumption were related to the macronutrients’ postprandial metabolic responses. It was observed that both postprandial serum triglycerides and glucose after lipid and carbohydrate meals, respectively, are better tolerated when the meals are consumed in the earlier active phase and finished at least 3 h prior sleeping time [105]. Regarding protein consumption, the recommendations are toward its even distribution throughout the eating periods. Ketogenic diets were also investigated and showed some benefits for metabolic health, namely, glucose tolerance [102]. Additionally, some amino acids were investigated as supplements, including tryptophan (precursor for melatonin), serine, ornithine, and glycine, showing improvement in sleep quality and efficiency, shortened sleep latency and reduced wake-time fatigue [101].

Other investigated supplements showing some benefits on sleep/wake periods when the circadian rhythm is disrupted include melatonin, a well-known chronobiotic which improves sleep and is often prescribed as a “sleeping pill”. Creatine was found to increase alertness and help during lack of sleep episodes when taken earlier during the active phase and with the first meal consumed. Caffeine, critical for alertness and cognitive functions (especially in shift workers), is not discouraged, but is recommended in smaller doses; every 2 h with the last consumption at least seven hours before the sleep time [101]. The adherence to these recommendations obviously depends on each individual’s preferences and work/family structure, as well as social, economic and other lifestyle circumstances.

Physical performance, dependent on muscle strength, power, contractibility and endurance, is also subject to diurnal variations, the lowest generally being in the morning and the most optimal in late afternoon [106]. However, these outcomes are also dependent on the individual’s chronotype, meaning that for the “morning-type person” the optimal performance will be reached earlier in a day and vice versa [107]. In this context, the environmental temperature plays a role too; hot temperatures may blunt muscle performance at any time and for any chronotype [108]. It is worth noting that these normal variations may also be changed in cases of scheduled training (usually happens in the morning hours), as shown recently in athletes; resistance training at different times of day for 12 weeks shifted normal–high amplitudes in a way that training in the morning enhanced morning muscle power even in evening chronotypes [109].

Regarding shift workers, it was shown earlier that 15 min of a cycling event every hour during shift work was helpful in adjusting the circadian rhythm [110]. However, practicality issues as to how effectively this regimen could be implemented in real life and in a real work situation is uncertain and thus dampens the relevance of this recommendation. Other studies imply that exercise performed in the earlier hours of biological day time (the active phase) is more beneficial than when performed in later hours toward sleeping time (resting phase) [111], which is opposite compared to the optimal performance of individuals with an aligned circadian rhythm [102].

## 7. Summary and Conclusions

Again, it needs to be emphasized that because of the lack of longer-term primary studies in COVID-19 patients (either acute or recovered) or interventions regarding OSA, this discussion is based on existing knowledge, scientific hypotheses and observations derived from similar conditions or emerging studies just being published at the time of writing this paper.

**Figure 1:**
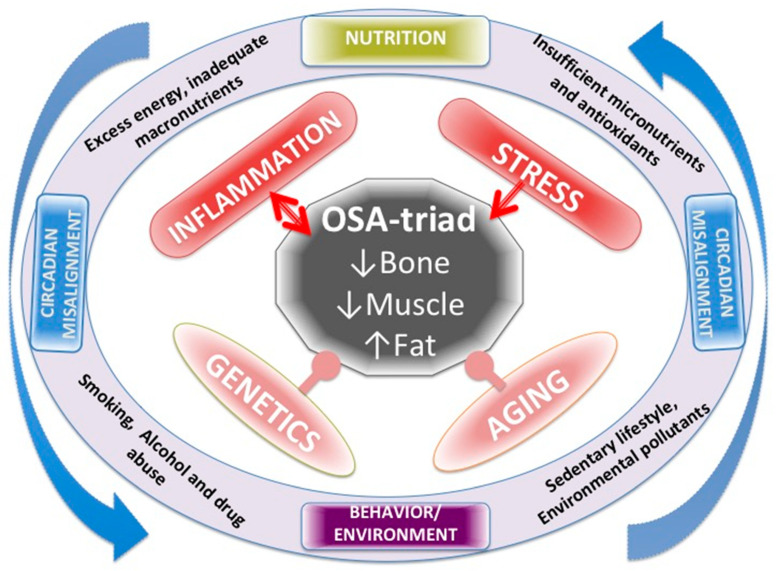
Various factors affecting body composition, presented as osteosarcopenic adiposity (OSA) and low-grade chronic inflammation (LGCI) as well as their mutual action on each other.

Figure 1 summarizes all the factors affecting OSA, including LGCI as well as their mutual action on each other. Genetics and aging are both inevitable and the most powerful influences on body composition and cannot be manipulated or changed. Chronic inflammation and stress are strong influences, but modifiable to a certain extent. Nutrition and lifestyle and all their impacts could be modified to a greater extent, including chronobiology and the circadian rhythm, when appropriate measures are taken, as has been addressed above.

Overall, within precision nutrition, maintaining appropriate energy intake, in order to retain a desirable weight and provide other nutrients, is especially important in the COVID-19 pandemic. Adequate macronutrient distribution should enable the right amount of good-quality protein, fiber and complex carbohydrate (with a minimal amount of simple sugars) and moderate fat intake (mostly in the form of mono and polyunsaturated fatty acids). The intake of vitamins, minerals and bioactive food components is emphasized in this period, as they provide essential nutrients for all metabolic processes, maintenance of all aspects of body composition, as well as defensive mechanisms against inflammation and viral infections.

Among vitamins, antioxidative vitamins, such as carotenoids (vitamin A precursors), and vitamins C, D and E have shown the most benefit when taken within the recommended levels. This is also the case for minerals, with particular emphasis on minerals that influence bone and body composition and are typically consumed in lower amounts, such as calcium, magnesium and potassium, and minerals with antioxidative and anti-inflammatory properties, such as copper, selenium and zinc, although a great caution should be taken when the supplemental amounts of all of those vitamins and minerals are taken.

Some bioactive food components, such as curcumin and quercetin, have shown the ability to obstruct coronaviruses by binding to the same cell receptor [82] and some have shown beneficial effects on both bone and muscle health. However, uncontrolled supplements are not advised due to their potential adverse effects, or no benefits [83]. The human microbiome has also received a lot of attention, especially with a view that it may reflect the presence of COVID-19 or other viruses [87,88] or undergo significant changes in the elderly when placed in nursing homes, leading to OSA [89]. Treatment with pre- and probiotic supplements could be considered to improve or enrich the microbiome, as well as provide beneficial effects on inflammation and ultimately body composition. Physical activity and exercise are now becoming a major issue, when gyms are closed and even some usual and habitual everyday activities are reduced. For older adults, any type of activity, including household chores, is considered beneficial [96], as well as alternate activities such as yoga, tai chi, or even ballroom dancing [97,98].

**Figure 2:**
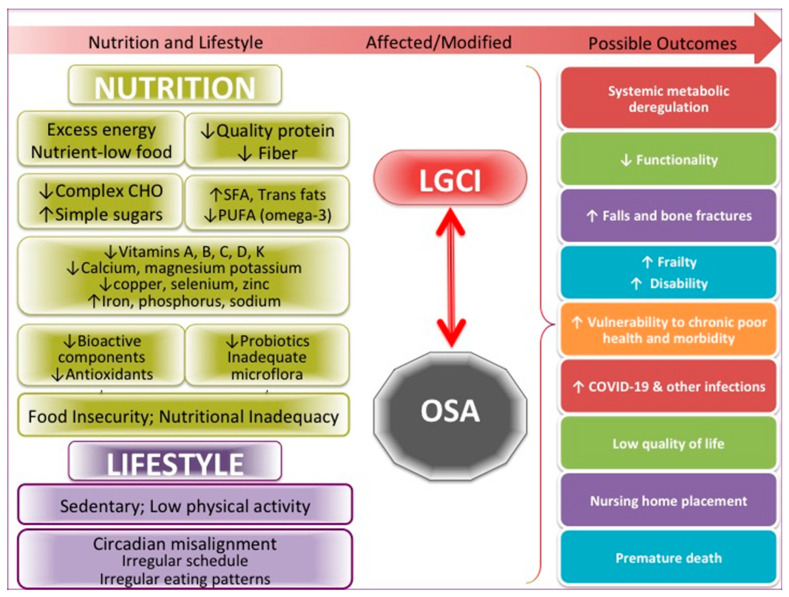
Specific nutritional and lifestyle factors affecting osteosarcopenic adiposity (OSA) and low-grade chronic inflammation (LGCI), leading to the increased risk for multiple adverse outcomes, including COVID-19. SFA, saturated fatty acids; PUFA, polyunsaturated fatty acids.

The many critical issues in this pandemic in the US, including food insecurity and nutritional inadequacy, also unproportionally affecting the elderly [9,99], deserve full attention on a national level in order to curb not just OSA but also many other comorbidities and prevent their convergence with COVID-19 infection. Misalignment in chronobiology caused by the explosion of the need for shift and night-time workers is another problem to be fully considered on a national level. Misalignment of the circadian rhythm and all the changes in food intake and usual activities that occur as a result may lead to numerous physiological and emotional consequences, as addressed above. Figure 2 summarizes the more specific factors, as have been discussed above, influencing both OSA and LGCI.

Currently, the duration of the COVID-19 pandemic is uncertain, but its consequences on human health are enormous. In addition to the death toll, evidence shows that many survivors end up with multiple problems affecting almost all organ systems, including bone, muscle and adipose tissue. There is even a new term referring to this condition as “long COVID” [112], or to people with lasting symptoms as “long haulers”. Many of the studies discussed here were conducted rapidly and more research is necessary to establish clear connections and causes/effects for some issues. However, waiting for the results of clinical trials or following the usual path of building up from basic science research to translation to animals, to humans, to patients and finally to community before providing treatments to patients or advising the general public (usually the most appropriate and scientifically sound way) is not feasible at this time. Therefore, health professionals depend on their ingenuity, experience and accumulated knowledge to manage patients and convey messages to the public. In cases where no clear damage is foreseen, many suggested lifestyle changes or supplemental therapies with nutrients (in cases of deficiencies) could be helpful and should be implemented.
